# Using Augmented Reality with Older Adults in the Community to Select Design Features for an Age-Friendly Park: A Pilot Study

**DOI:** 10.1155/2020/8341034

**Published:** 2020-09-01

**Authors:** Edgar R. Vieira, Fernanda Civitella, Jorge Carreno, Miburge G. Junior, Cesar F. Amorim, Newton D'Souza, Ebru Ozer, Francisco Ortega, Jansen A. Estrázulas

**Affiliations:** ^1^Department of Physical Therapy, Florida International University, 11200 SW 8^th^ St, AHC3-430, Miami, FL, USA; ^2^Department of Physical Therapy, Federal University of Sergipe, Av. Marechal Rondon, São Cristóvão, SE 49100, Brazil; ^3^Department of Physical Therapy, Sao Paulo City University, Rua Cesario Galeno, São Paulo, SP 44803071, Brazil; ^4^Department of Interior Architecture, Florida International University, 11200 SW 8^th^ Street, PCA 387b, Miami, FL 33199, USA; ^5^Department of Landscape Architecture + Environmental and Urban Design, Florida International University, 11200 SW 8^th^ St., PCA 374A, Miami, FL 33199, USA; ^6^Department of Computer Science, Colorado State University, 1873 Campus Delivery, Fort Collins, CO 80523-1873, USA; ^7^College of Health, Amazonas State University, 1777 Av. Carvalho Leal, Manaus, AM 69065, Brazil

## Abstract

Sedentary behavior is prevalent in older adults. Older adults often underutilize public parks for exercising because the parks do not support their needs and preferences. Engaging older adults on the redesign of parks may help promote active lifestyles. The objectives of this pilot study were to evaluate (1) the effects of wearing augmented reality (AR) and virtual reality (VR) glasses on balance; (2) the effects of different virtual walls separating the walking trail from the roadway on older adults' gait, and (3) the preferences of the participants regarding wall design and other features. The participants were ten older adults (68 ± 5 years) who lived within two miles from the park. Balance and gait were assessed using a force plate and an instrumented mat. It was feasible to use AR with older adults in the park to evaluate features for redesign. Motion sickness was not an issue when using AR glasses, but balance was affected when wearing VR goggles. The area of postural sway increased approximately 25% when wearing AR glasses, and it increased by close to 70% when wearing VR goggles compared to no glasses. This difference is clinically relevant; however, we did not have enough power to identify the differences as statistically significant because of the small sample size and large variability. Different walls did not significantly affect the participants' gait either because they did not alter the way they walked or because the holograms were insufficiently realistic to cause changes. The participants preferred a transparent wall rather than tall or short solid walls to separate the park from the roadway.

## 1. Introduction

Sedentary behavior is prevalent among older adults in the community; a systematic review of the literature found that 67% of older adults were sedentary for more than 8.5 hours/day [1]. Being active is crucial for older adults to sustain mobility and physical function. According to the Centers for Disease Control and Prevention (CDC), 7 out of 10 deaths occur among Americans per year due to chronic diseases, many of which are preventable by living an active and healthy lifestyle [2]. Environments that support physical activity in the communities where older people live are essential for active aging and are particularly important for older adults of lower socioeconomic status that cannot afford gyms and other activities that require a membership, equipment, and/or gear [3].

The design of public parks often does not support the needs or consider the preferences of older adults [4–6]. In addition, due to access impediments and poor upkeep, public parks are often underutilized by older adults [4]. Engaging older adults on the design or redesign of public spaces can be an effective way to promote the use of the spaces and active lifestyles [5]. Increased use of public spaces may result in higher levels of physical activity, socialization, improved health, and quality of life [6].

The use of virtual reality (VR) and augmented reality (AR) for visualization, education, and gaming environments is well documented [7–9]. VR and AR have also being used extensively to enhance exercise programs and in rehabilitation [10–12]. These technologies also have the potential to be used to engage older adults in public space design. However, because of its high degree of immersion, one needs to be cautious about using VR with older adults as it may lead to motion sickness, impair their gait and balance, and thereby threaten their safety [13–15]. One study found that the dynamic balance was significantly affected by wearing a head-mounted VR display; larger, faster, and longer center of pressure displacements were observed when people were wearing a head-mounted VR display showing the same scenes versus when being on the actual physical environments [16]. This is of concern because older adults have increased risk of falls and fall-related injuries [17].

AR may be an alternative to VR to assist in the visualization and selection of designs for public spaces since it is more amenable to older adults because of the lower degree of immersion that allows them to perceive the real space and position of their body segments through the overlaid holograms [15]. However, very few studies have used AR to engage older adults in community design such as the design of public parks. Therefore, it is important to evaluate the feasibility of doing that. In addition, the effects of AR on the users' gait and balance need to be further evaluated to assess if using this technology also affects older adults' gait and balance such as VR, potentially increasing the risk of falls.

In addition to need to evaluate the potential effects of AR itself on gait and balance, we found no studies assessing the effects of different AR conditions on older adults' gait and balance [18, 19]. In other words, we found no studies using AR with older adults to simulate different environmental conditions and evaluate how those conditions would potentially affect the users' gait and balance. Therefore, there is also need to evaluate how different design features presented using AR may affect the participants' gait and balance because the physical environment of parks, streets, and traffic barriers can affect older adults' mobility [20]. Actually, the physical environment can directly affect people's gait and balance. For example, older adults with stroke have been found to walk 8.8 m/min (95% CI: 0.3–17.3) faster in a clinic than in a mall (*P*=0.046) [21]. Potential explanations for the difference include the environmental design (e.g., walls) and purpose of walking (shopping vs. medical assessment). The tasks performed and the physical environmental features of the location where the tasks are performed affect balance by modifying the input information used for postural control [22]. Therefore, different traffic barriers between roadways and pedestrian pathways may affect the pedestrians' balance and gait. Physical environments affect gait and balance, and the risk of falls in older adults [23]. A reduction of excessive environmental exposures (e.g., using walls to restrict the sight of moving cars and noise) may improve balance and gait by reducing distractions and decrease the risk of falls. However, little is known about the potential impacts of walking path characteristics (e.g., pedestrian/cyclist separation, lighting, and signage) and street traffic barriers' design (e.g., walls) displayed using AR on older adults' gait and balance.

Wall traffic barriers can increase safety for pedestrians and reduce noise. Actual and perceived risks associated with traffic in close proximity to walking paths discourage walking and cycling, especially in high traffic speed areas [24]. Perceived safety is a critical aspect for pedestrians in judging how walkable a public space and a city is [25]. Walls are often used as traffic barriers for increased safety and noise reduction, especially to separate motor vehicles from pedestrians in adjacent parks and recreational and residential areas [26–28]. Short walls/barriers (often two feet high), tall wall barriers, and, more recently, transparent wall barriers are often used to separate traffic from parks and recreational and residential areas to increase safety and reduce noise [29]. The type and characteristic of the physical barriers separating traffic from pedestrians affect perceived safety and may affect users' mobility (e.g., gait and balance) [30, 31]. However, the effects of walls separating traffic from pedestrians in adjacent parks need to be further evaluated.

Taking into account older user preferences for the design of parks and public spaces may increase use and safety. AR may be a suitable technology to engage older adults in the process. However, further studies are required to evaluate this potential. Considering the reasons presented in this introduction, the objectives of this pilot study were as follows:

1. To evaluate the effects of wearing AR and VR glasses on older adults' gait and balance
2. To assess the effects of different public park design features on older adults' gait and balance
3. To evaluate older adults' preferences regarding physical environment features for the redesign of a public park

We labeled the study as a pilot because of how innovative it is. We were not sure if the field-based combination of AR and VR with gait and balance assessment in a public park would work, nor if it would support the evaluation of the user's design preferences. Another reason why we labeled it as a pilot is because we enrolled only ten participants.

## 2. Materials and Methods

The project was a collaboration between researchers from physical therapy, landscape and interior architecture, and computer science. The investigations were carried out following the rules of the Declaration of Helsinki of 1975 (https://www.wma.net/what-we-do/medical-ethics/declaration-of-helsinki/), revised in 2013. According to point 23 of this declaration, an approval from an ethics committee was obtained before undertaking the research. The study protocol was submitted and approved by the institutional review board of the Florida International University Office of Research Integrity (protocol #18-107186). All participants signed an informed consent form.

The methodology involved (1) evaluating the effects of AR and VR on the participants' balance using force places (the gold-standard equipment for balance assessment); (2) evaluating the effects of AR on the participants' gait spatiotemporal parameters using a GAITRite (an instrumented mat for gait analysis); (3) evaluating the effects of changing AR-displayed wall barriers on the participants' gait using the same equipment, and (4) assessing how much the participants liked the different characteristics displayed using Likert scales (also a gold-standard method for assessing preferences).

### 2.1. Participants

The participants were ten older adults (68 ± 5 years) who lived in the community within two miles from a public park. The inclusion criteria were age ≥60 years, living within two miles of the park, be English or Spanish speakers, and ability to walk independently (without assistive devices such as canes or walkers) for a block, have no lower limb surgery or injuries from falls during the previous 6 months, and pass the Mini-Cog test. The participants were recruited from a local government facility where neighborhood meetings are held, and by word of mouth (friends and neighbors of the participants). We recruited older adults who lived in close proximity to the park because they are familiar with the site and would benefit the most from having a redesigned park with their preferences taken into account. They are the people who would be most interested in redesigning the space according to their preferences. These are underserved older adults living in an impoverished neighborhood suffering greatly from gentrification. They are community dwelling older adults that can ambulate independently. They were paid $10 for their time, but we believe that the strongest motivation for participation was the hope to be part of ideas for redesigning the park.

### 2.2. Procedures

Testing was conducted outdoors in a public park adjacent to a main highway with no barrier between traffic and the walking paths. The participants walked at their preferred pace on an instrumented mat (GAITRite®, SN: Q209, CIR Systems Inc). The GAITRite® system measures of speed, cadence, step length, and step time were found to have high concurrent validity (ICC = 0.91–0.99) when compared to Vicon-5121 system measures [32]. Also, the reliability of repeated measures of single and double support times using the GAITRite® system was found to be high (ICC = 0.85–0.93) [33]. In general, the reliability of these system measures of temporospatial parameters of gait has been found to be excellent for both young and older adults [34]. The participants completed a familiarization walking trial followed by three recording trials under each condition in random order. The following gait parameters will be assessed:

- Velocity, walking speed in cm/s calculated as distance covered divided by the ambulation time
- Cadence, the number of steps per minute
- Step length, distance between the heel center of 1 foot to the heel center of other foot during heel strike
- Step width, the distances between a line linking the center of 1 foot during 2 subsequent steps and the center of the opposite foot during midstance

The participants walked on the mat under the following conditions:

1. Not wearing AR glasses (*no wall*)
2. Wearing AR glasses (Microsoft's HoloLens) displaying design features including a *tall wall* between the highway and the park, lane separators for bikes and pedestrians, benches, light fixtures, a bathroom, and additional vegetation
3. Wearing AR glasses displaying a *short wall* between the highway and the park and the other features
4. Wearing AR glasses displaying a *transparent wall* between the highway and the park and the other features

**Figure 1:**
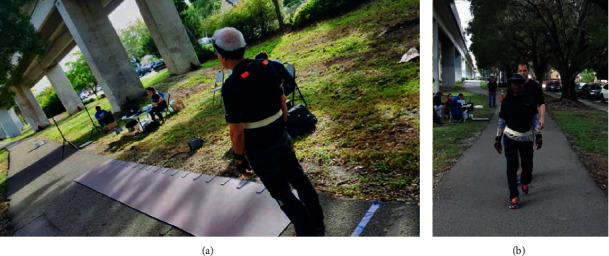
Participant at the beginning of the mat before initiating a walking trial (a) and a participant finishing the walk after crossing the mat (b).

**Figure 2:**
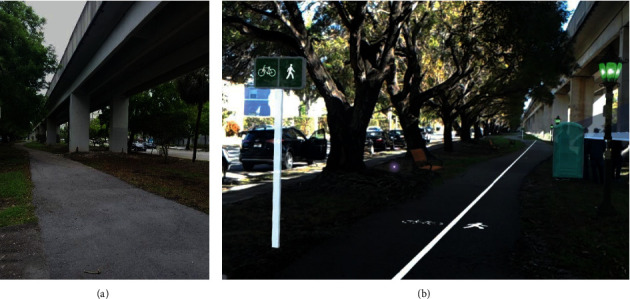
Environment not including (a) and including (b) the augmented reality displays of (from right to left) a transparent wall, light fixtures, bathroom, lane separations for bikes and pedestrians, benches, and signage.

These wall types were selected because we wanted to check the security and safety perception of older adults with different degrees of transparency and height. Participants were free to stop participating at any time.  Figure 1 displays a participant positioned at the beginning of the mat before initiating a walking trial (a) and a participant finishing the walk after crossing the mat (b).  Figure 2 displays the environment not including (a) and including (b) the AR displays of a transparent wall, lane separations for bikes and pedestrians, signage, lights, benches, and bathroom.

Balance was assessed based on the oscillation of the center of pressure and its positioning tendency. The following variables were analyzed using a portable force plate (AMTI OR6-700: 502 × 502 × 45 mm) with 32-bit digital data transmission, acquisition rate of 1000 Hz for 3 channels (Mx, My, and Mz), including a fixed 3^rd^ order 100 Hz analog filter and analysis software (AMTI® Accugait Balance Clinic): 95% elliptical area of postural sway/center of pressure displacement while standing still, laterolateral (*X*) and anteroposterior (Y) velocity, and acceleration of the center of pressure. The AMTI® system provides quantitative measures of static and dynamic balance performance and visual feedback of the excursion and position of the center of pressure.

**Figure 3:**
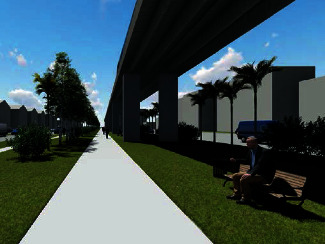
Fully immersive virtual reality environment used for balance testing.

The participants stood up on the force plate looking forward for 20 seconds without glasses, then wearing AR glasses, and then with VR goggles (HTC Vive) displaying the same environment in full immersion (Figure 3). We included VR to do a direct comparison with AR during the quasistatic balance tests. Considering the current popularity of VR systems, city planners may want to use VR. Therefore, we included it to evaluate changes in balance compared to AR and no glasses/goggles. The computer scientists who are part of the team and co-authors designed, produced, and coded the AR and VR environments.

The participants completed one familiarization trial followed by two testing trials. We evaluated standing balance with both AR and VR, but we did not include a VR condition for the gait assessment due to the previously established risks for falls when walking while wearing VR [13–15].

**Figure 4:**
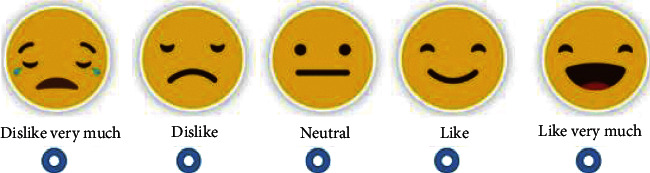
Pictorial scale used to score the design features displayed as augmented reality holograms.

After the walking and balance trials, the participants indicated how much they liked or disliked each of the features from “dislike very much” to “like very much” (Figure 4). After the selections, the participants were asked “Are there any other features that you would like to see in place that would further encourage you to use this space?” Their responses were audio-recorded and noted down.

### 2.3. Data Analysis

The gait parameters under the different conditions were normalized by the values obtained when walking without glasses (control condition). The effects of the conditions on gait speed, cadence, step length, and base of support were compared using ANOVAs. The balance measures while wearing AR glasses and VR goggles were normalized by the measures taken when not wearing them (control condition). Differences in balance between the conditions (AR vs. VR) were assessed using Student's *t*-tests. All tests were done using SPSS 18 at a significance level of 0.05. The participants' responses regarding the different features and traffic barriers were presented descriptively as percentages, and the comments from the participants were paraphrased and quotes were presented for illustration.

## 3. Results

Neither wearing AR glasses nor the display of different design features affected the participants' gait significantly. There were no statistically significant differences among the conditions for any of the gait variables: velocity (no holograms = 114 cm/s, short wall = 119 cm/s, tall wall = 118 cm/s, and transparent wall = 117 cm/s; *P*=0.480); cadence (no holograms = 109 steps/min, short and tall wall = 112 steps/min, and transparent wall = 111 steps/min; *P*=0.446); step length (all conditions = 63 cm; *P*=0.499); base of support (no holograms, short and tall wall = 10 cm, and transparent wall = 11 cm; *P*=0.433).  Table 1 presents the actual and normalized data by the results obtained when walking without AR glasses.

The center of pressure variables during the balance testing with AR and VR are presented in Table 2. The data are presented as a percentage of the control condition (no glasses/goggles).

The 95% elliptical area of center of pressure sway increased approximately 25% when wearing AR glasses and close to 70% when wearing VR goggles (mean difference = 41%). The difference was borderline (a *P* value of 0.06 was close to the 0.05 cutoff), but it did not reach statistical significance given the high variability. Statistically significant differences were found only for laterolateral (side-to-side) velocity (mean difference = 24%). Clinically meaningful differences were also found for anteroposterior velocity (mean difference = 24%) [35, 36]. On the other hand, acceleration of the center of pressure was not affected by wearing either goggle.

Table 3 indicates how much the participants liked or disliked each of the features displayed using AR holograms over the existing environment.

The participants tended to prefer the transparent wall as a traffic barrier; 90% of the participants marked that they “liked” or “liked very much” the transparent wall. Seventy percent of the participants “liked” or “liked very much” the small wall, and 40% “liked” or “liked very much” the tall wall. On the other hand, 40% “disliked very much” the tall wall; they mentioned that they were afraid that the tall wall would limit visibility and increase the risk of violence or robbery because other people would not see them in the park in case a “bad guy” approached them. They also commented that the additional vegetation should not be tall so that it would not “provide hiding places” (personal safety concerns).

All participants approved the lane separation; 100% “liked” or “liked very much” the bike/pedestrian lane separation, as well as the benches. Similarly, 90% “liked” or “liked very much” the lampposts and bathroom. Seventy percent of the participants “liked” or “liked very much” the signage, while 30% said they were neutral (neither liked nor disliked). Additional features that the participants said they would like to see in place on the park included exercise equipment, water fountains, food and beverage vendors, a dog park (off-leash space), trees and flowers/landscaping (but no hiding places, safety concern), bike racks, trash bins, and distance/mile markers.

## 4. Discussion

This was a small study using AR with older adults outdoors in a public park (the first one to do it). We evaluated the effects of using AR and VR on the balance of the users; we evaluated the effects of using AR on the gait of the users. We also evaluated the effects of different AR-displayed walls on the participants' gait and balance, and evaluated the participant's ratings of the different walls, lane separators for bikes and pedestrians, benches, light fixtures, a bathroom, and vegetation/trees features using Likert scales. We believe that this study contributes to the advancement of the field. We established that AR can be safely used with older adults in field studies and identified their preferences for the redesign of the park. Future, public space designers may involve older adults using AR in a similar way to increase participation and account for users' preferences. VR is increasingly being used to design and redesign houses and roads [37]. AR is not used as often, but its popularity and application is increasing; further adoption is likely but dependent of cost-benefit analysis studies [38].

We were able to collect balance and gait data during the outdoor field-testing. We compared three types of walls (small, tall, and transparent) to separate the park from the roadway. It was feasible and useful to use AR with older adults in the community to evaluate different design features for the redesign of a public park to encourage an active lifestyle among older adults. Chan et al. [39] evaluated spatiotemporal gait parameters of treadmill walking in three conditions: (1) control, (2) AR, and (3) VR, and found a significant difference in stride length and cadence between the control and AR conditions. In contrast, we found neither wearing AR glasses nor the display of different design features significantly affected the participants' gait on a park pathway. The results indicate either that the different wall conditions did not affect how the participants walked or that the holograms were insufficiently realistic to cause changes. Future evaluations need to test the effects of physical mockups versus hologram simulations of environmental characteristics on older adults' gait. Some of the issues encountered and lessons learned include the fact that the number of features displayed/holograms need to be small; contrast can be an issue with sunlight, and brighter and lighter colors show more evidently in the AR holograms when used outdoors in the daylight. We had to adapt the initial holograms to reduce transparency by using brighter (white) colors for the wall holograms to decrease transparency and distinguish between them.

Motion sickness was not an issue for any of the participants when using AR glasses, but balance was affected when wearing VR goggles. The change in balance was clinically relevant; however, we did not have enough power to identify the differences as statistically significant because of the small sample size and large variability among subjects. The area of postural sway increased approximately 25% when wearing AR glasses compared to no glasses, and it increased by close to 70% when wearing VR goggles. These findings are in agreement with Horlings et al. [40], which also found that use of VR goggles increased the postural sway and sway velocity of subjects in quiet stance, on both firm and compliant surfaces. This shows that AR did not disturb balance very much, but that fully immersive virtual reality impaired standing balance and needs to be used with caution, especially in older adults who already experience increased postural instability due to decreases in vestibular and proprioceptive function [7, 41].

The use of AR holograms provided insights into older adult preferences based on interviews and surveys conducted with ten older adults regarding design feature selections before they are built. Identifying features that are preferred by older adults is needed so that the ongoing redesign of the space can include the stated preferred characteristics. The participants are becoming advocates for the redevelopment and use of the space. The results are being presented and used to advocate for evidence-based redesign of the park to make it more age-friendly. The evidence-based redesign of the space, accounting for local older adults' preferences, should support increased use by this population.

This study was conducted with the context of the Miami-Dade Underline Project (https://www.theunderline.org/); our study was funded through a minigrant program sponsored by the Miami-Dade County Age-Friendly Initiative (https://agefriendlymiami.org/). The findings were shared with the Underline team and are being used in the redesign of the area. The underline project includes members of the community and of the Miami-Dade Age-Friendly Initiative. These members are aware of the findings of our study and are actively engaged in selecting and approving the plans for redesigning the park. A redesigned park including the preferred features is likely to result in increased utilization. Future studies are needed to evaluate this aspect. Increased use may result in increased levels of physical activity and socialization improving quality of life and health for older adults in the area [6]. The participants were enthusiastic about using AR to visualize options for an existing environment, which is noteworthy for a future design and redesign of public spaces.

## 5. Conclusions

It was feasible and useful to use AR with older adults in the community to evaluate different environmental features for the redesign of a public park to encourage an active lifestyle among older adults. The results indicate either that the different wall conditions did not affect how the participants walked or that the holograms were insufficient to cause real changes. Wearing AR glasses had a small effect on participants balance, but wearing VR goggles impaired the participants balance. The participants preferred a transparent wall design to a tall or short solid wall designs to separate the park from traffic on the adjacent roadway.

## Figures and Tables

**Table 1:** Gait parameters while walking with augmented reality (AR) glasses with no holograms and with different types of wall. Mean ± standard deviation of the actual values and as percentage increase from the trial without AR glasses between parentheses.

	No holograms	Short wall	Tall wall	Transparent wall	*F*	*P*
Velocity in cm/s (%)	114 ± 22 (2 ± 5)	119 ± 25 (4 ± 8)	118 ± 24 (3 ± 6)	117 ± 27 (2 ± 9)	0.21	0.89
Cadence in steps/min (%)	109 ± 7 (1 ± 3)	112 ± 9 (3 ± 5)	112 ± 10 (2 ± 4)	111 ± 11 (1 ± 5)	0.46	0.71
Left step length in cm (%)	63 ± 11 (1 ± 3)	63 ± 11 (1 ± 5)	63 ± 10 (1 ± 5)	62 ± 11 (0 ± 5)	0.24	0.87
Right step length in cm (%)	63 ± 10 (0 ± 3)	63 ± 10 (1 ± 4)	63 ± 10 (0 ± 3)	64 ± 11 (1 ± 5)	0.10	0.96
Left step width in cm (%)	10 ± 2 (3 ± 15)	10 ± 3 (0 ± 17)	10 ± 3 (8 ± 14)	12 ± 3 (22 ± 20)	2.39	0.09
Right step width in cm (%)	10 ± 2 (2 ± 19)	10 ± 3 (7 ± 19)	10 ± 2 (7 ± 15)	11 ± 3 (22 ± 14)	2.39	0.09

**Table 2:** Comparison of balance of older adults when wearing augmented reality (AR) glasses and virtual reality (VR) goggles. Data normalized by the values obtained when not wearing them (control condition).

Center of pressure variable	AR	VR	*P*
95% elliptical area	126 (32)	167 (72)	0.061^∼^
Anteroposterior velocity	98 (33)	122 (41)	0.191^∼^
Laterolateral velocity	101 (20)	125 (35)	0.047^*∗*^
Anteroposterior acceleration	101 (4)	102 (8)	0.372
Laterolateral acceleration	98 (9)	106 (6)	0.095

^∼^Not statistically significant, but a clinically meaningful difference [35, 36]; ^*∗*^*p* < 0.05.

**Table 3:** Percentage of the 10 participants that assigned each rating to each feature.

	Dislike very much (%)	Dislike (%)	Neural (%)	Like (%)	Like very much (%)
Transparent wall	0	10	0	30	60
Small wall	10	10	10	50	20
Tall wall	0	40	20	10	30
Lane separation	0	0	0	20	80
Lamp posts	0	0	10	10	80
Benches	0	0	0	30	70
Bathroom	0	0	10	20	70
Signage	0	0	30	30	40

## Data Availability

Data are available on request through the authors.
